# Depression in breast cancer patients who have undergone mastectomy: A national cohort study

**DOI:** 10.1371/journal.pone.0175395

**Published:** 2017-04-10

**Authors:** Min-Su Kim, So Young Kim, Jin-Hwan Kim, Bumjung Park, Hyo Geun Choi

**Affiliations:** 1 Department of Otorhinolaryngology-Head and Neck Surgery, CHA Bundang Medical Center, CHA University, Seongnam, Korea; 2 Department of Otorhinolaryngology-Head & Neck Surgery, Hallym University College of Medicine, Seoul, Korea; 3 Department of Otorhinolaryngology-Head & Neck Surgery, Hallym University College of Medicine, Anyang, Korea; Iranian Institute for Health Sciences Research, ISLAMIC REPUBLIC OF IRAN

## Abstract

**Objective:**

The objective of this study was to compare the incidence of post-operative depression in breast cancer patients who have undergone mastectomy with the incidence of post-operative depression in non-breast cancer participants (controls).

**Methods:**

Using data from the Korean Health Insurance Review and Assessment Service (HIRA), we selected 2,130 patients with breast cancer who have undergone mastectomy for this national cohort study and matched these patients 1:4 with 8,520 control participants according to age, sex, income, region, and pre-operative depression. The incidence of post-operative depression was measured from mastectomy year to post-op year 10. The Mann-Whitney U test was used for data analysis, and the false-discovery rate was applied to determine statistical significance (P < 0.05).

**Results:**

The incidence of depression was higher in the breast cancer with mastectomy group than in the control group up to 3 years after mastectomy). However, there was no difference in the incidence of depression between the breast cancer with mastectomy group and the control group after post-op 4 years. The incidence of depression was higher in the breast cancer with mastectomy group than in the control group up to 2 years after mastectomy, and there was no difference in the incidence of depression between the two groups after post-op 3 years in middle-aged and older adults (≥ 40 years old). In young adults (≤ 39 years old), the incidence of depression was significantly higher in the breast cancer with mastectomy group than in the control group in mastectomy year.

**Conclusion:**

Patients undergoing mastectomy for breast cancer experience depression more frequently than healthy people. However, patients overcome their depressive mood symptoms during the postoperative period. Young adults overcome their symptoms more quickly than middle-aged and older adults.

## Introduction

Breast cancer is the most common invasive cancer in women worldwide, affecting approximately 12% of women [1]. Breast cancer accounts for 22.9% of invasive cancers in women and 16% of all female cancers [2]. A Korean study reported that breast cancer accounted for 14.8% of all female cancers in 2012 [3]. In recent years, the incidence of breast cancer has increased because the population has aged. However, the 5-year-survival rate for breast cancer has improved due to earlier tumor detection and improvements in treatment. These developments have resulted in increasing numbers of women surviving breast cancer [4]. These women experience a wide range of functional and emotional impairments, such as depression, after mastectomy, and these impairments have profound psychosocial effects. Studies in Western countries have demonstrated that the prevalence of depression after mastectomy ranges from 1% to 56% [5]. Depression can be relieved by appropriate treatment, which has been demonstrated to be highly effective [6]. A randomized controlled trial (RCT) reported that the Center for Epidemiologic Studies Depression Scale scores of 100 patients were significantly improved from 25.4 to 14.8 by psychoeducational intervention [7], and another RCT demonstrated that Hamilton Depression Rating Scale scores were decreased by 7.51 by cognitive behavioral therapy [8]. However, major depression and depressive symptoms tend to be undertreated in women with breast cancer, which may be due to the fact that women with breast cancer are generally reluctant to disclose information regarding changes in their affects or emotional states or that oncologists are not familiar with depressive symptom screening [9]. Mood disorder diagnosis failure can be problematic because depression and its associated symptoms diminish quality of life and affect compliance with medical therapies and may reduce survival [10].

In studies in which the long-term effects of breast cancer on depression were analyzed, the effect of time since diagnosis was almost never considered [4, 11, 12]. To our knowledge, participants of most studies were lesser than our study [5]. The purpose of this study was to compare the incidence of post-operative depression in patients with breast cancer who have undergone mastectomy with the incidence of post-operative depression in non-breast cancer participants (control) using data from a national cohort study. In this study, we matched patients with breast cancer who underwent mastectomy with control subjects at a 1:4 ratio according to age, sex, income, region, and pre-operative depression. We followed up these participants for a minimum of 1 year and a maximum of 10 years.

## Materials and methods

### Study population and data collection

The ethics committee of Hallym University (2014-I148) approved the use of the indicated data. The requirement of written informed consent was waived by the Institutional Review Board of Hallym University.

This national cohort study used data from the Korean Health Insurance Review and Assessment Service—National Patient Sample (HIRA-NPS). The Korean National Health Insurance Service (NHIS) selected data directly from the entire population database to prevent non-sampling errors. Data pertaining to approximately 2% (one million) of the entire Korean population (50 million) were selected. These data were classified into 1,476 levels (age [18 categories], sex [2 categories], and income levels [41 categories]) via proportional allocation using randomized stratified systematic sampling methods to represent the entire population. After data selection, the appropriateness of the sample was verified by a statistician who compared data from the entire Korean population with the sample data. The details regarding the methods used for these procedures were described by the National Health Insurance Sharing Service [13]. The cohort database included (i) personal information, (ii) health insurance claim codes (procedures and prescriptions), (iii) International Classification of Disease-10 (ICD-10) diagnostic codes, (iv) socio-economic data (residence and income), and (v) medical examination data for each participant for the period ranging from 2002 to 2013.

As all Korean citizens have a 13-digit resident registration number from birth to death, exact population statistics can be obtained from the above database. It is mandatory for all Koreans to enroll in the NHIS, and all Korean hospitals and clinics use the 13-digit resident registration number to register individual patients in the medical insurance system. Therefore, the risk of patient medical record overlap is minimal, even if a patient is transferred to another facility for further treatment. Moreover, all medical treatments in Korea can be tracked using the HIRA system. Korean law requires that all deaths are reported to an administrative entity before funerals can be held, and causes of death are recorded by medical doctors on death certificates.

### Participant selection

**Fig 1:**
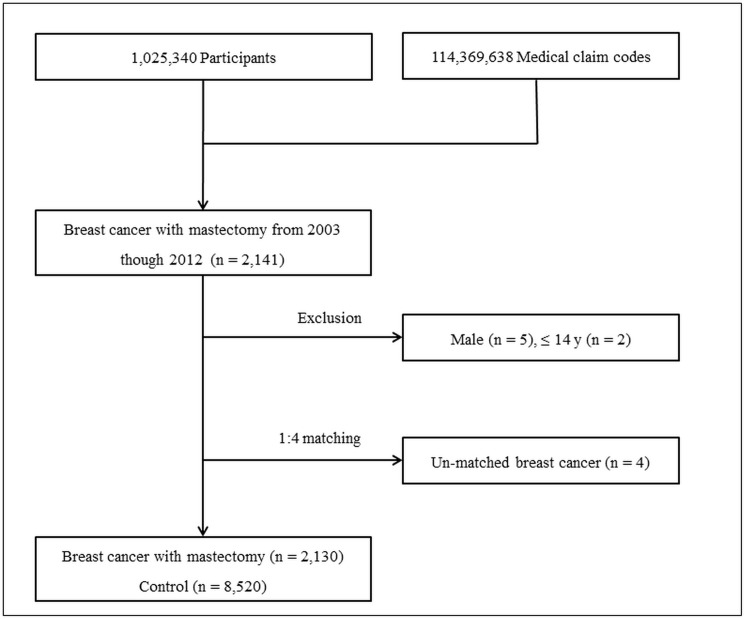
Schematic illustration of the participant selection process of the present study. The indicated number of breast cancer patients who underwent mastectomies from 2003 to 2012 were selected from a total of 1,025,340 patients (n = 2,141). After removal of unmatched participants, male participants, and participants younger than 15 years old, 2,130 participants were ultimately included in the study. These participants were matched 1:4 with 8,520 control participants.

The indicated number of participants (n = 2,141) underwent mastectomy (claim codes: N7131-N7135) for breast cancer (ICD-10: C50) from 2003 to 2012, were selected from 1,025,340 cases with 114,369, 638 medical claim codes and were included in this study. Male participants (n = 5) and participants younger than 15 years old (n = 2) were excluded from the study. The breast cancer with mastectomy participants were matched 1:4 according to age, income, region of residence and the number pre-operative depression episodes for 1 year with participants who did not undergo mastectomy from 2002 to 2013 and were never diagnosed with breast cancer. Control participants were selected in 1,025,340 participants. To prevent selection bias when selecting the control group, control subjects were randomly sorted before being selected from top to bottom. Participants for whom an insufficient number of matching participants was found were excluded (n = 4). Ultimately, 2,130 breast cancer with mastectomy patients and 8,520 control subjects were enrolled in the study (Fig 1).

### Variables

**Fig 2:**
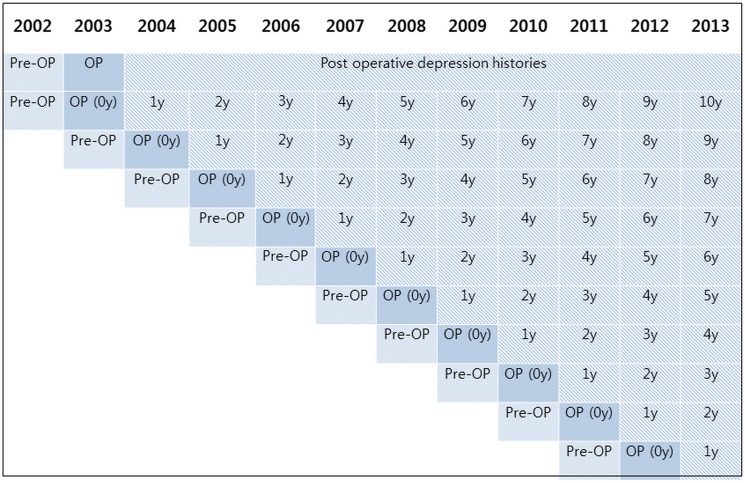
Schematic illustration of participant depression histories according to the time elapsed since mastectomy for breast cancer.

The participants were organized into age groups separated by 5-year intervals as follows: 15–19, 20–24, 25–29…, and 85+ years, resulting in the creation of 15 age groups. The income groups were initially divided into 41 classes (one health-aid class, 20 self-employment health insurance classes, and 20 employment health insurance classes), which were subsequently were re-organized into 11 classes (class 1 [lowest income]-11 [highest income]). Regions of residence were divided into 16 areas, according to administrative district, before being reorganized into urban (Seoul, Busan, Daegu, Incheon, Gwangju, Daejeon, and Ulsan) and rural areas (Gyeonggi, Gangwon, Chungcheongbuk, Chungcheongnam, Jeollabuk, Jeollanam, Gyeongsangbuk, Gyeongsangnam, and Jeju). Depression histories were defined using ICD-10 codes (F31), and the numbers of clinic and hospital visits for depression were counted each year. Pre-operative depression histories were counted for 1 year, and post-operative depression histories were counted each year of the study (1 y, 2 y, 3 y….10 y) (Fig 2).

To compare the depression histories of breast cancer participants who died during follow up with those of matched control subjects, only control subjects whose depression history durations corresponded with the survival durations of matched breast cancer patients were used for analysis (S1 Table).

### Statistical analyses

Because the data regarding the numbers of depression histories exhibited a near-power law distribution rather than a normal distribution, we performed the Mann-Whitney U test to analyze the differences between the two groups. It was difficult to conceptualize the differences between the case-control groups using the Mann-Whitney U test; therefore, data pertaining to the case-control groups were expressed as the mean and standard deviation (SD).

Because we performed the Mann-Whitney U test 11 times (post-op year 0 to post-op year 10) in the same analysis, we applied false-discovery rates to prevent type I errors. Two-tailed analyses were conducted, and the adjusted statistical significance was set as 0.05. The results were analyzed using SPSS v. 21.0 (IBM, Armonk, NY, USA).

For subgroup analysis, the participants were divided into following 2 groups: young adults (≤ 39 years old) and middle-aged and older adults (≥ 40 years old).

## Results

Because the groups were matched, they were similar with respect to general patient characteristics (age group, sex, income level, region of residence) (Table 1).

**Table 1:** General participant characteristics.

Characteristics		Number of participants			
		Breast cancer	Control group	Total participants	% of column
Age (years old)					
	15–19	9	36	45	0.4
	20–24	25	100	125	1.2
	25–29	131	524	655	6.2
	30–34	216	864	1,080	10.1
	35–39	356	1,424	1,780	16.7
	40–44	460	1,840	2,300	21.6
	45–49	356	1,424	1,780	16.7
	50–54	209	836	1,045	9.8
	55–59	170	680	850	8.0
	60–64	99	396	495	4.6
	65–69	61	244	305	2.9
	70–74	22	88	110	1.0
	75–79	11	44	55	0.5
	80–84	4	16	20	0.2
	85+	1	4	5	0.0
Income					
	1 (lowest)	26	104	130	1.2
	2	125	500	625	5.9
	3	133	488	610	5.7
	4	148	592	740	6.9
	5	161	644	805	7.6
	6	188	752	940	8.8
	7	217	868	1,085	10.2
	8	210	840	1,050	9.9
	9	280	1,120	1,400	13.1
	10	312	1,248	1,560	14.7
	11 (highest)	341	1,364	1,705	16.0
Region of residence					
	Urban	1,142	4,568	5,710	53.6
	Rural	988	3,952	4,940	46.4

We compared the numbers of visits for depression in the mastectomy group with the numbers of visits for depression in the control group during the follow-up period. The numbers of visits for pre-operative depression were exactly same in both groups. The incidence of depression was higher in the breast cancer with mastectomy group than in the control group up to 3 years after mastectomy [(Post-op year 0 (P < 0.001), 1 (P < 0.001), 2 (P = 0.002), and 3 (P = 0.014)]. However, there was no significant difference of depression after 4 years of mastectomy (Table 2).

**Table 2:** The numbers of patients with pre-operative and post-operative depression histories in the breast cancer and control groups.

	Breast cancer (mean, SD)	Control (mean, SD)	P-value
Pre-op depression (n = 10,650)	0.05 ± 0.44	0.05 ± 0.44	1.000
Post-op 0 y depression (n = 10,650)	0.14 ± 0.81	0.10 ± 1.04	< 0.001*
Post-op 1 y depression (n = 10,650)	0.19 ± 1.24	0.14 ± 1.42	< 0.001*
Post-op 2 y depression (n = 9,035)	0.19 ± 1.37	0.15 ± 1.38	0.002*
Post-op 3 y depression (n = 7,410)	0.21 ± 1.41	0.17 ± 1.42	0.014*
Post-op 4 y depression (n = 6,075)	0.22 ± 1.57	0.21 ± 1.61	0.828
Post-op 5 y depression (n = 5,095)	0.17 ± 1.15	0.22 ± 1.96	0.183
Post-op 6 y depression (n = 4,085)	0.23 ± 1.52	0.20 ± 1.47	0.738
Post-op 7 y depression (n = 3,005)	0.15 ± 0.95	0.20 ± 1.54	0.233
Post-op 8 y depression (n = 2,150)	0.29 ± 1.59	0.23 ± 1.64	0.052
Post-op 9 y depression (n = 1,475)	0.44 ± 2.44	0.26 ± 1.88	0.587
Post-op 10 y depression (n = 745)	0.67 ± 2.99	0.29 ± 2.00	0.077

* Mann-Whitney U test. False-discovery-rate-adjusted significance: P < 0.05.

Among young adults (≤ 39 years), the incidence of depression was significantly higher in the breast cancer group than in the control group in a mastectomy year and 8 years of mastectomy [(Mastectomy year (P < 0.001) and 8 years of mastectomy (P = 0.001)]. Among middle-aged and older adults (≥ 40 years), the incidence of depression was significantly higher in the breast cancer group than in the control group until 2 years after mastectomy [(Mastectomy year (P < 0.001), 1 (P = 0.001), and 2 (P = 0.017)] (Table 3).

**Table 3:** Subgroup analysis of the numbers of patients with pre-operative and post-operative depression histories in the breast cancer and control groups.

	Breast cancer (mean, SD)	Control (mean, SD)	P-value
**Age ≤ 39 years old**			
Pre-op depression (n = 3,685)	0.05 ± 0.38	0.05 ± 0.38	1.000
Post-op 0 y depression (n = 3,685)	0.12 ± 0.77	0.09 ± 0.93	< 0.001*
Post-op 1 y depression (n = 3,675)	0.17 ± 1.23	0.12 ± 1.27	0.057
Post-op 2 y depression (n = 2,975)	0.20 ± 1.51	0.10 ± 1.03	0.033
Post-op 3 y depression (n = 2,255)	0.13 ± 1.11	0.12 ± 1.21	0.137
Post-op 4 y depression (n = 1,755)	0.20 ± 1.89	0.13 ± 1.29	0.710
Post-op 5 y depression (n = 1,400)	0.13 ± 1.02	0.10 ± 0.94	0.341
Post-op 6 y depression (n = 1,060)	0.28 ± 2.24	0.10 ± 0.85	0.903
Post-op 7 y depression (n = 745)	0.17 ± 1.27	0.12 ± 1.24	0.329
Post-op 8 y depression (n = 475)	0.33 ± 1.78	0.07 ± 0.71	0.001*
Post-op 9 y depression (n = 320)	0.23 ± 1.39	0.10 ± 1.20	0.845
Post-op 10 y depression (n = 135)	0.00 ± 0.00	0.06 ± 0.43	0.478
**Age ≥ 40 years old**			
Pre-op depression (n = 6,965)	0.05 ± 0.47	0.05 ± 0.47	1.000
Post-op 0 y depression (n = 6,965)	0.15 ± 0.83	0.11 ± 1.10	< 0.001*
Post-op 1 y depression (n = 6,930)	0.20 ± 1.24	0.15 ± 1.50	0.001*
Post-op 2 y depression (n = 6,060)	0.19 ± 1.30	0.18 ± 1.51	0.017*
Post-op 3 y depression (n = 5,155)	0.25 ± 1.52	0.20 ± 1.50	0.042
Post-op 4 y depression (n = 4,320)	0.22 ± 1.42	0.24 ± 1.72	0.679
Post-op 5 y depression (n = 3,695)	0.19 ± 1.19	0.27 ± 2.23	0.308
Post-op 6 y depression (n = 3,025)	0.21 ± 1.17	0.24 ± 1.63	0.759
Post-op 7 y depression (n = 2,260)	0.14 ± 0.82	0.23 ± 1.63	0.377
Post-op 8 y depression (n = 1,675)	0.28 ± 1.53	0.28 ± 1.82	0.492
Post-op 9 y depression (n = 1,155)	0.49 ± 2.17	0.30 ± 2.03	0.612
Post-op 10 y depression (n = 610)	0.82 ± 2.46	0.34 ± 2.20	0.046

* Mann-Whitney test. False-discovery-rate-adjusted significance: P < 0.05.

## Discussion

This study demonstrated that the incidence of depression was higher in the mastectomy group than in control group up to 3 years after mastectomy. Therefore, mastectomy affect post-operative depression during only 3 years. However, we did not observe a difference in the incidence of depression between the mastectomy and control groups after 4 years of mastectomy (Tables 2 and S1). A French cohort study demonstrated that the emotional gap between breast cancer survivors and population-based control subjects seems to narrow as the time interval since the onset of breast cancer increases [14]. In that study, breast cancer survivors exhibited significantly poorer emotional symptom scores than control subjects at 5 years post-breast cancer onset. The differences in symptom scores between the two groups decreased with time, as breast cancer survivors exhibited higher emotional symptom scores at 10 and 15 years post-breast cancer onset than at 5 years post-breast cancer onset and were no longer different from control subjects with respect to their symptom scores. However, that study analyzed emotional symptoms at 5-year intervals, whereas our study analyzed depressive symptoms annually [14]. One systematic review demonstrated that the prevalence of depression symptoms was higher among breast cancer survivors than among the general female population at one year after diagnosis and that increases in the time since diagnosis exerted positive effects on psychological wellbeing similar to those described above [15]. Some authors attributed this phenomenon to adjustment processes for breast cancer survivors, noting that depressive symptoms occurred during the first 4 years post-cancer onset and improved with time [16]. The above findings may also be attributed to the fact that most breast cancer recurrences and treatments, such as invasive examinations, operations, radiation treatments, and chemotherapy treatments, occur within the first 3 years after diagnosis [17]. However, although the durations of depressive symptoms were different between mastectomy patients and control subjects in the above studies, the frequencies of depression were similar between mastectomy patients and control subjects in all the studies after recovery from breast cancer [14–17].

Our study demonstrated that depression durations were shorter in young adults (≤ 39 years old) than in middle-aged and older adults (≥ 40 years old). A previous Danish population-based cohort study demonstrated that older age was associated with depression in 44,494 women with breast cancer [18]. Hung et al also noted an increased risk for mood disorders among older women in a group of 26,629 women with breast cancer who were followed up to 7 years after diagnosis [19]. Older women suffer from co-morbidities, such as hypertension, diabetes mellitus, chronic obstructive pulmonary disease, ischemic heart disease, and cerebrovascular disease, which are independent risk factors for depression. Many studies have demonstrated that these factors are associated with depression and interfere with recovery from depression [20–25]. Among young adults, the incidence of depression was higher in the breast cancer group than in the control group at 8 years after mastectomy, a difference that seemed to reflect statistical variation rather than statistical significance.

One advantage of this study was its large number of participants (N = 10,650). We followed up the patients who underwent mastectomies for a maximum period of 10 years, whereas most previous studies followed up patients who underwent mastectomies for 5 years [10, 11, 26, 27]. An additional advantage of this study was the availability of comprehensive patient medical records for each participant. Previous studies obtained information regarding participant histories of depressive symptoms via self-reporting, which may have resulted in recall bias [14, 28]. However, in this study, we used patient medical records and HIRA data to obtain information regarding depression histories and visits. These recorded data were not affected by patient memory. Moreover, HIRA data were available for the entire nation, without exception. Therefore, we did not lose any participants to follow-up, whereas other studies lost significant numbers of patients to follow-up. Although we did not use RCT methods, we matched our participants with control subjects according to age, sex, income, region of residence, and previous depression visits. Income and region matching were important, as these variables can be determining factors with respect to the types of medical care and services utilized by patients. Income levels were determined very accurately using the Korean NHIS, as patient premiums were determined based on patient incomes. Our study results were representative of the entire Korean population, as our data were selected from a database covering the entire population, and their representativeness was verified by a statistician.

However, our study had several limitations. We used health insurance claim data and counted the numbers of visits for depression, which may not have been reflective of the actual numbers of depressive episodes experienced by patients. Using ICD codes in the large claim code could have the possibility of misdiagnosis. However, the medical claim is very important in Korea, because the diagnosis affect the medical doctor and participants in various ways. If the claim codes is not correct, the medical claim fee could be cut by the national health insurance services. The drug for depression, anti-depressant, could not be prescribed to patient without the exact diagnosis codes. The patients could be rejected to the private insurance. This clam codes data do not have the possibility of recall bias. Therefore, the medical diagnosis would be more accurate than survey or other subjective data in other studies. Moreover, the present study design compensate the possibility of misdiagnosis, even though some of patient were misdiagnosed. We used the patient-control studies with the large participants, considering the possibility of misdiagnosis would exist in both patient (breast cancer) and control group.

Additionally, we believe that the numbers of visits for depression can serve as a surrogate index of the numbers of depressive episodes in studies involving large amounts of data. We could not measure depression severity in each participant, and the numbers and types of services utilized by each patient could vary, even among patients with the same disease. Additionally, it was difficult to compare results between the two groups because many of the participants had no depression history. Moreover, it was not clear whether patients’ depressive symptoms were caused by their breast cancers or their mastectomies.

## Conclusions

The results of our study suggest that women who have undergone mastectomy for breast cancer have a higher risk for developing short-term (≤ 3 years) depression symptoms than women in the general population. Moreover, middle-aged and older breast cancer patients are at risk for longer depression durations after mastectomy than younger patients. Early appropriate psychiatric referrals may enable the identification of patients with depression and thus facilitate the provision of appropriate psychosocial support and medical aid to improve patient quality of life.

## Supporting information

S1 Table (DOCX)
