# Supplementary material for: Depression in breast cancer patients who have undergone mastectomy: A national cohort study
Source: PLoS One. 2017 Apr 10;12(4):e0175395. doi: 10.1371/journal.pone.0175395 (PMC5386257; doi:10.1371/journal.pone.0175395)
Supplement: S1 Table — (DOCX) [file pone.0175395.s001.docx]

S1 Table. Comparison of depression between breast cancer and control group

|  | | Breast cancer (n, %) | Control (n, %) | P-value |
| --- | --- | --- | --- | --- |
| Pre-op depression | |  |  | 1.000 |
|  | Yes | 51 (2.4%) | 204 (2.4%) |  |
|  | No | 2,079 (97.6%) | 8,316 (97.6%) |  |
| Post-op 0y depression | |  |  | < 0.001* |
|  | Yes | 118 (5.5%) | 212 (2.5%) |  |
|  | No | 2,012 (94.5%) | 8,308 (97.5%) |  |
| Post-op 1y depression | |  |  | < 0.001* |
|  | Yes | 101 (4.8%) | 262 (3.1%) |  |
|  | No | 2,020 (95.2%) | 8,222 (96.9%) |  |
| Post-op 2y depression | |  |  | 0.002* |
|  | Yes | 80 (4.4%) | 215 (3.0%) |  |
|  | No | 1,727 (95.6%) | 7,013 (97.0%) |  |
| Post-op 3y depression | |  |  | 0.013* |
|  | Yes | 65 (4.4%) | 183 (3.1%) |  |
|  | No | 1417 (95.6%) | 5745 (96.9%) |  |
| Post-op 4y depression | |  |  | 0.819 |
|  | Yes | 50 (4.1%) | 193 (4.0%) |  |
|  | No | 1,165 (95.9%) | 4,667 (96.0%) |  |
| Post-op 5y depression | |  |  | 0.169 |
|  | Yes | 45 (4.4%) | 143 (3.5%) |  |
|  | No | 974 (95.6%) | 3,933 (96.5%) |  |
| Post-op 6y depression | |  |  | 0.759 |
|  | Yes | 37 (4.5%) | 140 (4.3%) |  |
|  | No | 780 (95.5%) | 3,128 (95.7%) |  |
| Post-op 7y depression | |  |  | 0.214 |
|  | Yes | 30 (5.0%) | 93 (3.9%) |  |
|  | No | 571 (95.0%) | 2,311 (96.1%) |  |
| Post-op 8y depression | |  |  | 0.050 |
|  | Yes | 26 (6.0%) | 67 (3.9%) |  |
|  | No | 404 (94.0%) | 1,653 (96.1%) |  |
| Post-op 9y depression | |  |  | 0.629 |
|  | Yes | 16 (5.4%) | 56 (4.7%) |  |
|  | No | 279 (94.6%) | 1,124 (95.3%) |  |
| Post-op 10y depression | |  |  | 0.084 |
|  | Yes | 12 (8.1%) | 27 (4.5%) |  |
|  | No | 137 (91.9%) | 569 (95.5%) |  |

* Pearson Chi-square test. Significance at false discovery rate adjusted P < 0.05
